# AQP4‐ab‐Positive Neuromyelitis Optica Spectrum Disorder Increases the Risk of Hydrocephalus: A Bidirectional Mendelian Randomization Study

**DOI:** 10.1002/brb3.70804

**Published:** 2025-10-15

**Authors:** Weitao Zhong, Weisong Li, Qiwei Huang, Zelin Li, Qiang Wang, Wangming Zhang

**Affiliations:** ^1^ Neurosurgery Center, Guangdong Provincial Key Laboratory on Brain Function Repair and Regeneration Zhujiang Hospital Southern Medical University Guangzhou China

**Keywords:** AQP4, hydrocephalus, Mendelian randomization, neuromyelitis optica spectrum disorder

## Abstract

**Introduction**: Some observational studies indicated that AQP4‐ab‐positive neuromyelitis optica spectrum disorder (NMOSD) may predispose to hydrocephalus. However, the causal relationship between NMOSD and hydrocephalus remains elusive. We used bidirectional Mendelian randomization (MR) to examine the causal effect of AQP4‐ab‐positive NMOSD and hydrocephalus.

**Methods**: The exposure GWAS data used in this study were obtained from the GWAS Catalog, which included 132 AQP4‐ab‐positive patients and 1244 normal controls. The outcome GWAS data for hydrocephalus (*N*__case_ = 2455, *N*__control_ = 382,198) were obtained from FinnGen R10. We used the inverse‐variance weighted (IVW) method to perform the principal analyses. Then, we used the Cochrane *Q*‐statistics test to assess the presence of heterogeneity and MR‐Egger‑intercept test to evaluate the pleiotropy for sensitivity analyses. A reverse MR analysis was used to investigate the potential for reverse causation.

**Results**: In the IVW analysis, we found that genetically predicted AQP4‐ab‐positive NMOSD was significantly associated with the increasing risk of hydrocephalus (OR = 1.05; 95% CI: 1.02–1.08; *p *= 7.65 × 10^−5^). In reverse MR analysis, we did not find genetically predicted hydrocephalus significantly associated with AQP4‐ab‐positive NMOSD (*p *> 0.05). In the sensitivity analysis, both the primary and reverse MR results exhibit no heterogeneity and horizontal pleiotropy.

**Discussion**: Our results indicate that genetically predicted AQP4‐ab‐positive NMOSD significantly increases the risk of hydrocephalus. The reduced immune activity of AQP4 may play an important role in the pathogenesis of hydrocephalus.

## Graphical abstract

This study employed Mendelian randomization (MR) analysis to investigate the causal relationship between AQP4‐antibody‐positive neuromyelitis optica spectrum disorder (NMOSD) and hydrocephalus. Using genetic variants as instrumental variables, we analyzed data from the GWAS Catalog (*N*_case = 132, *N*_control = 1244) for AQP4‐positive NMOSD exposure and FinnGen R10 (*N*_case = 2455, *N*_control = 382,198) for hydrocephalus outcomes. The analysis utilized inverse‐variance weighted methods as the primary approach, with sensitivity analyses including reverse MR, the Cochrane *Q*‐statistics test, and the MR‐Egger intercept to assess the robustness of causal inferences and potential pleiotropy effects.

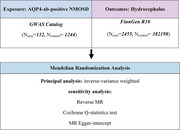

## 1 Introduction

Neuromyelitis optica spectrum disorder (NMOSD) is a central nervous system autoimmune inflammatory disease, which is divided into two groups of patients based on antibody status: AQP4‐ab‐positive and AQP4‐ab‐negative subgroups (Wingerchuk et al. 2015; Wingerchuk and Lucchinetti 2022). The AQP4 antibodies bind to AQP4 on perivascular astrocytic endfeet, thereby attenuating the immunoreactivity of AQP4 in the central nervous system of AQP4‐ab‐positive NMO patients (Y. Guo et al. 2017). This reduction appears to be closely related to hydrocephalus, as accumulating evidence suggests that decreased AQP4 activity is associated with the onset and exacerbation of hydrocephalus (de Laurentis et al. 2021; Bloch et al. 2006; J. Guo et al. 2018; Mestre et al. 2018; Feng et al. 2009). Inhibition of AQP4 expression may exacerbate the severity of kaolin‐induced and autologous blood‐induced hydrocephalus in the rodent (Bloch et al. 2006; J. Guo et al. 2018). Additionally, brain biopsy results indicated a significant reduction in AQP4 on the perivascular astrocytic endfeet in idiopathic normal pressure hydrocephalus patients compared to normal brain tissue from epilepsy patients (Hasan‐Olive et al. 2019). Moreover, a growing body of case reports and series have documented the emergence of hydrocephalus subsequent to NMOSD (Clardy et al. 2014; Chiba et al. 2017; Close et al. 2019). An observational study conducted by the Mayo Clinic suggests that patients with NMOSD have a significantly higher incidence of hydrocephalus compared to the general population in California (Clardy et al. 2014; Patwardhan and Nanda 2005). Interestingly, according to these observational studies, the type of hydrocephalus subsequent to NMOSD includes obstructive hydrocephalus (Chiba et al. 2017) and communication hydrocephalus (Close et al. 2019).

These observational studies show a clear chronological sequence, indicating that AQP4‐ab‐positive NMOSD with AQP4 dysfunction may predispose to hydrocephalus. We hypothesize that AQP4‐ab‐positive NMOSD increases the risk of hydrocephalus. However, conclusions drawn from traditional observational studies may be prone to coincidence due to biases and confounding factors. Furthermore, the relatively low incidence rate of NMOSD (0.37‐10/100,000) (Papp et al. 2021) and ethical restrictions pose considerable challenges in the implementation of randomized controlled trials (RCTs). Therefore, we employ a Mendelian randomization (MR) study to validate this hypothesis.

MR provides a unique advantage in this setting by utilizing summary‐level genetic data from large‐scale genome‐wide association studies (GWAS), enabling us to explore potential causal relationships even for rare diseases. MR utilizes single‐nucleotide polymorphism (SNP) loci for causal association assessment. Allele genes are randomly assigned during genetic processes, similar to the random grouping in an RCT. Given that individual genotypes remain constant throughout the lifespan, MR analysis can mitigate interference from confounding factors and other reverse causation biases (Davies et al. 2018). The effectiveness of MR analysis is equivalent to an RCT, with the added advantages of practicality and convenience. Herein, we applied a bidirectional MR analysis to investigate the causal effects of genetically predicted AQP4‐ab‐positive NMOSD on hydrocephalus. Our research aims to provide novel perspectives on the role of AQP4 in the pathology of hydrocephalus and to present novel considerations for nonsurgical therapy of hydrocephalus.

## 2 Methods

### 2.1 Study Design

Our research used the two‐sample MR design to investigate the causal relationships between NMOSD and hydrocephalus. Our MR study follows the three key evaluations: (1) the instrument variants are closely related to the exposure. (2) Both the instrument variants and the outcome are not affected by confounding factors. (3) The instrument variants affect the outcome through the exposure without any direct effects.

### 2.2 Data Source

The exposure data used in this study were obtained from the previous AQP4‐ab‐positive NMOSD GWAS, which included 132 AQP4‐ab‐positive patients and 1244 normal controls (AQP4 antibody seropositivity confirmed by ELISA or cell‐based assay) (Estrada et al. 2018). The outcome data for hydrocephalus (*N* __case_ = 2455, *N* __control_ = 382,198) were obtained from FinnGen R10 (Kurki et al. 2023). All the GWAS data are based on European ancestry.

### 2.3 Selection of Instrumental Variants

Instrumental variables were chosen based on the following three principles: (1) SNPs are not in linkage disequilibrium, defined as *r* ^2 ^< 0.1 and a distance of 10,000 kb (Yan et al. 2024; Huang et al. 2020); (2) genome‐wide significance of the SNPs meets the threshold *p *< 5 × 10^−8^. Then we extracted the IVs from the outcome data. The effect allele from both exposure and outcome datasets was harmonized. (3) F‐statistic 〉 10 to ensure all the instruments are strongly associated with the exposure and to exclude weak instrumental variables (Pierce et al. 2011). We excluded the SNPs that exist the palindromic sequence. We utilized the LDtrait tool provided by the NIH (https://ldlink.nih.gov/?tab=ldtrait) to examine SNPs associated with the primary outcome in our analysis, addressing potential confounding factors that might violate assumption 2. To meet assumption 3, we excluded SNPs significantly associated with the outcomes (*P *< 5 × 10^−8^).

### 2.4 Statistical Analysis

The principal MR analysis was conducted using the inverse‐variance weighted (IVW) method. We conducted three sensitivity analyses to assess the robustness of the MR result. We used the MR‐Egger intercept test to identify potential horizontal pleiotropy and the MR‐PRESSO global test to remove the outlier SNPs. Cochrane *Q*‐statistics were used to assess the presence of heterogeneity, and a Cochran's *Q p* < 0.05 was regarded as an indication of heterogeneity. Additionally, we performed a reverse using hydrocephalus as an exposure and AQP4‐ab‐positive NMOSD as an outcome to explore reverse causation between AQP4‐ab‐positive NMOSD and the AQP4‐ab‐positive NMOSD. The selection of instrumental variants and analysis methods for this reverse MR analysis is consistent with the description above.MR analyses and plotting were conducted using the “TwoSampleMR” package of the R software package (version 4.3.0). Statistical significance was considered at a 2‐sided *p *< 0.05.

## 3 Result

**FIGURE 1:**
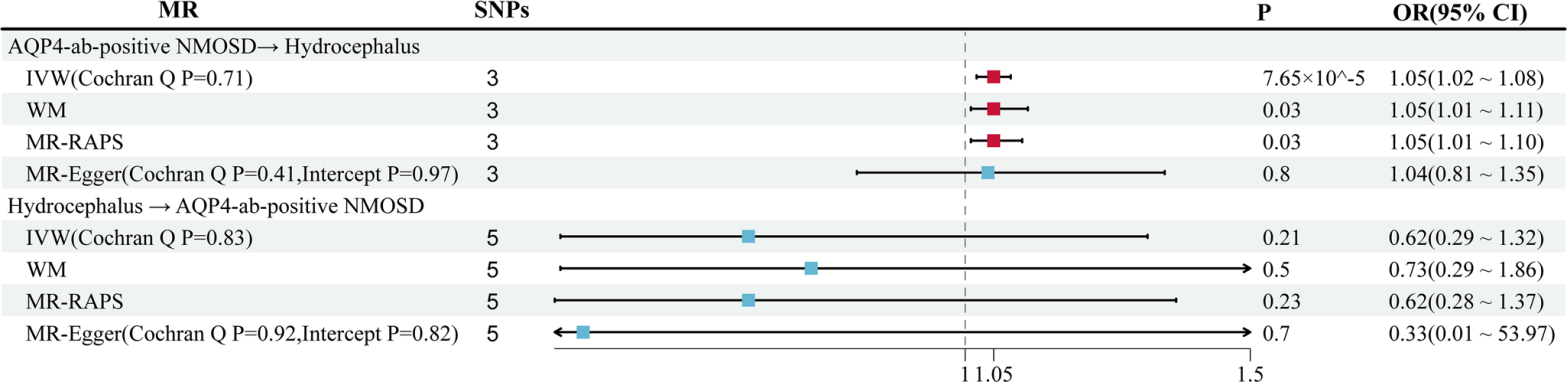
MR results for the associations of AQP4‐ab‐positive NMOSD with hydrocephalus and the reverse MR results.

**FIGURE 2:**
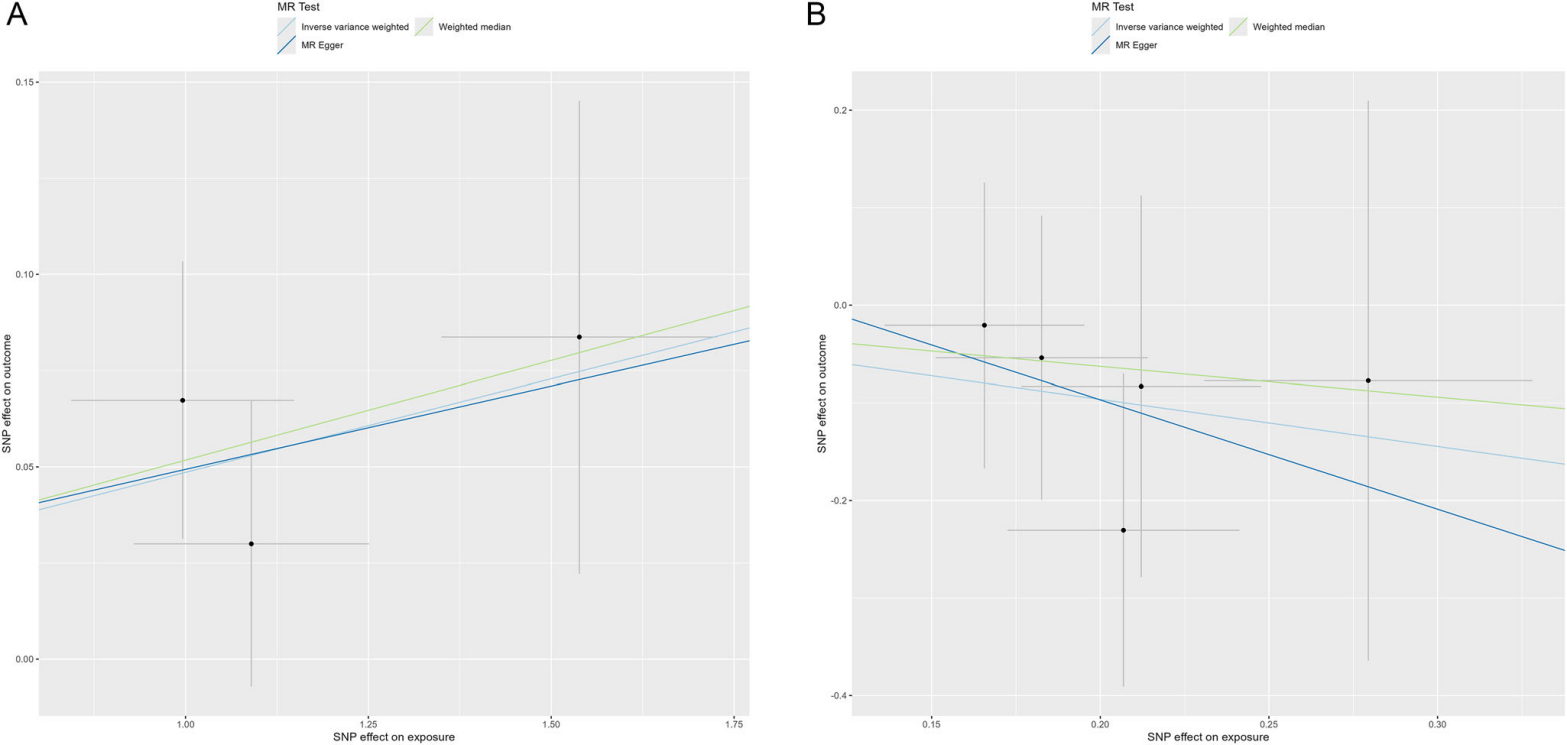
Scatter plot of the MR analysis results for the effect of genetically predicted AQP4‐ab‐positive NMOSD on hydrocephalus and the reverse MR analysis results. A. The effect of genetically predicted AQP4‐ab‐positive NMOSD on hydrocephalus B. The effect of genetically predicted hydrocephalus on AQP4‐ab‐positive NMOSD.

In the primary MR analysis, we finally selected three SNPs from AQP4‐ab‐positive NMOSD GWAS as instrumental variants; detailed information of all SNPs is presented in Table 1. The results of the MR analyses and sensitivity analysis between AQP4‐ab‐positive NMOSD and hydrocephalus are presented in Figure 1. In the IVW analysis, we found that genetically predicted AQP4‐ab‐positive NMOSD was significantly associated with the increasing risk of hydrocephalus (OR = 1.05; 95% CI: 1.02–1.08; *p *= 7.65 × 10^−5^). The MR‐Egger analysis and MR‐RAPS method also showed identical results (OR = 1.05; 95% CI: 1.01–1.11; *p* = 0.03; OR = 1.05, 95% CI: 1.01–1.10; *p *= 0.03). Besides, the direction of the effect in the results of the WM analysis method aligns with the two aforementioned methods (Figure 2). In the MR‐Egger intercept method for the pleiotropy test, we found no potential pleiotropy in the MR result. In the Cochrane *Q*‐statistics, we also found no heterogeneity (Figure 1).

**TABLE 1:** Detailed Information of instrumental variable.

SNP	Effect allele	Other allele	beta	se	*p*	*F*
AQP4‐ab‐positive NMOSD						
rs1150757	A	G	−1.5385	0.1885	3.33E−16	66.5999
rs616187	A	G	−0.9961	0.1524	6.34E−11	42.7118
rs9368716	A	G	1.0900	0.1610	1.27E−11	45.8587
Hydrocephalus						
rs12253527	A	G	−0.18261	0.0314	6.21E−09	33.7666
rs4579156	C	T	0.212137	0.0355	2.36E−09	35.6534
rs4820308	A	G	0.165654	0.0296	2.30E−08	31.2226
rs79424089	G	A	0.279457	0.0487	9.30E−09	32.9832
rs7962919	A	G	−0.20687	0.0344	1.87E−09	36.0998

In the reverse MR analysis, we finally selected five SNPs from the hydrocephalus GWAS as instrumental variants (Table 1). The results showed that genetically predicted hydrocephalus was not significantly associated with AQP4‐ab‐positive NMOSD (*p *> 0.05). In the sensitivity analysis, the MR results exhibit no heterogeneity and horizontal pleiotropy (Figure 1).

Figure 1 presents the causal estimates derived from bidirectional MR analyses using three complementary methods: IVW, weighted median (WM), MR‐RAPS, and MR‐Egger. Evaluates the causal effect of genetically predicted AQP4‐antibody‐positive NMOSD on the risk of hydrocephalus.

## 4 Discussion

To our knowledge, this is the first MR analysis to examine the causal relationship between AQP4‐ab‐positive NMOSD and hydrocephalus. Our study results demonstrate that genetically predicted AQP4‐ab‐positive NMOSD has a causal relationship with a higher risk of hydrocephalus. The sensitivity analysis was consistent with the primary results, affirming the stability and consistency of the MR analysis. Besides, we have not identified a reverse causal relationship between AQP4‐ab‐positive NMOSD and hydrocephalus.

AQP4 is a member of the aquaporin protein family, first discovered in 1994 (Hasegawa et al. 1994). In the central nervous system, AQP4 is predominantly expressed on the end feet of astrocytes that contribute to the blood–brain barrier (Verkman 2012), facilitating the selective transport of water in and out of cells (Mangiatordi et al. 2016) to regulate intracerebral fluid balance. The delicate intracerebral fluid balance, however, is disrupted in hydrocephalus—a disease characterized by the abnormal accumulation of cerebrospinal fluid (CSF) within the brain's ventricles and still no definitive cure (McAllister 2011, Del Bigio and Di Curzio 2016). AQP4 may play a crucial role in the pathophysiology of hydrocephalus (de Laurentis et al. 2021). Y. Guo et al. (2017) demonstrated that AQP4 immunoreactivity in AQP4‐ab‐positive NMOSD patients was reduced compared to control levels in the central nervous system, as observed through autopsies. Furthermore, the density and immunoreactivity of AQP4 on the endfeet of astrocytes are reduced in patients with idiopathic normal‐pressure hydrocephalus (iNPH) through a detailed comparison of the biopsy results from the cerebral cortex of iNPH patients and the control group (including epilepsy, tumor, and cerebral aneurysm patients) (Hasan‐Olive et al. 2019; Eide and Hansson 2018). Our MR analysis results did support the hypothesis that AQP4‐ab‐positive NMOSD increases the risk of hydrocephalus. Therefore, we can reasonably speculate that the decreased immune activity of AQP4 may be a significant factor contributing to the onset of hydrocephalus by integrating these autopsy findings (Y. Guo et al. 2017; Hasan‐Olive et al. 2019; Eide and Hansson 2018) with our MR analysis result. Consistent with our result, an increasing amount of research underscores the critical role of AQP4 in the pathogenesis of hydrocephalus. The experimental results in animal models of hydrocephalus indicate that silencing the expression of AQP4 or knocking out the AQP4 gene may contribute to hydrocephalus (Bloch et al. 2006; J. Guo et al. 2018; Mestre et al. 2018; Feng et al. 2009). What's more, restoring the expression of AQP4 has been shown to inhibit the development of hydrocephalus after experimentally induced perinatal intraventricular hemorrhage (Purohit et al. 2021). While accumulating evidence supporting the significant role of AQP4 in the pathogenesis of hydrocephalus in rodent models, exercising prudence is essential when directly extending this conclusion to humans, considering the differences in the distribution and regulation of AQP4 between rodents and the human brain (Skjolding et al. 2013). However, due to ethical constraints, direct exploration of the impact of AQP4 on the human body, such as silencing the expression of AQP4, is not feasible. AQP4‐ab‐positive NMOSD is a disease characterized by the presence of AQP4 antibodies and reduced AQP4 immunoreactivity (Wingerchuk et al. 2015; Wingerchuk and Lucchinetti 2022; Y. Guo et al. 2017). Therefore, AQP4‐ab‐positive NMOSD serves as a valuable substitute for investigating the role of AQP4 in the onset of hydrocephalus. Conducting MR analysis enabled the exploration of evidence supporting a causal effect of AQP4‐ab‐positive NMOSD on hydrocephalus. Simultaneously, evidence is available to establish whether the decline in AQP4 immunoreactivity is linked to an increased susceptibility to hydrocephalus. This is a highlight of our research, utilizing AQP4‐ab‐positive NMOSD as an exposure variable to investigate the role of AQP4 in the onset of hydrocephalus.

The causal association between AQP4‐ab‐positive NMOSD and hydrocephalus was consistent with previous observations. Three previous case reports showed unexplained hydrocephalus (including obstructive hydrocephalus and communication hydrocephalus) in patients with NMOSD (Clardy et al. 2014; Chiba et al. 2017; Close et al. 2019). The largest observational study about AQP4‐ab‐positive NMOSD and hydrocephalus to date, conducted by the Mayo Clinic in 2014, included 177 AQP4‐ab‐positive NMOSD patients. This study suggested that the incidence of hydrocephalus in AQP4‐ab‐positive NMOSD patients is approximately 1.7% (3/177) (Clardy et al. 2014). In contrast, the annual overall incidence of hydrocephalus is only 2.95 per 100,000 across all types in California, United States (Patwardhan and Nanda 2005). However, other population‐based cohorts did not report any hydrocephalus associated with NMOSD (Nagaishi et al. 2011; Viktoria et al. 2021). For example, a large Japanese cohort that included 583 AQP4‐ab‐positive NMOSD patients reported no occurrences of hydrocephalus among them; in contrast to the aforementioned studies, this study reported no occurrences of any hydrocephalus among these patients (Nagaishi et al. 2011). These studies primarily focus on the epidemiology and clinical characteristics of NMOSD, overlooking the documentation of hydrocephalus as a comorbidity. Consequently, they may not adequately illustrate the association between NMOSD and hydrocephalus. Nevertheless, the limitations of the studies mentioned above, such as small sample size and a cross‐sectional study design, could restrict the generalizability of establishing a causal relationship. Conclusions drawn from these traditional observational studies may be prone to coincidence. But a well‐designed, large‐scale, multicenter prospective study and randomized controlled trials are time‐consuming, resource‐intensive, and costly, particularly for rare diseases such as NMOSD (Papp et al. 2021; Viktoria et al. 2021). Therefore, there is still a deficiency in well‐designed prospective cohort studies to comprehensively uncover the association between NMOSD and hydrocephalus. The MR study emerges as a viable option for answering the question of whether AQP4‐ab‐positive NMOSD increases the risk of hydrocephalus. Our MR study results contribute significantly to elucidating the relationship between AQP4‐ab‐positive NMOSD and hydrocephalus.

Our study reveals a statistically significant but modest association between AQP4‐ab‐positive NMOSD and hydrocephalus risk (OR = 1.05). The modest OR aligns with clinical practice, where AQP4‐ab‐positive NMOSD patients exhibit a higher risk of developing hydrocephalus compared to the general population, though the absolute risk remains relatively low. Previous studies have reported that approximately 1.7% of NMOSD patients develop hydrocephalus (Clardy et al. 2014). Notably, although all AQP4‐ab‐positive NMOSD patients harbor pathogenic AQP4 antibodies, only a small subset develops hydrocephalus, suggesting that the pathogenesis involves complex, multifactorial mechanisms beyond simple antibody presence. The differential susceptibility to hydrocephalus among AQP4‐ab‐positive patients indicates that additional genetic, environmental, or disease‐specific cofactors may be required for clinical manifestation. Our findings provide a novel research perspective by highlighting the need to investigate what distinguishes NMOSD patients who develop hydrocephalus from those who do not. This approach may help elucidate the complex pathophysiological mechanisms underlying hydrocephalus development and potentially identify new biomarkers or therapeutic targets.

Our findings suggest that enhancing AQP4 activity and increasing AQP4 expression in the early stages of hydrocephalus development could attenuate disease progression. This aligns with the concept that maintaining optimal AQP4 function is crucial for proper CSF homeostasis. The AQP4 upregulation observed in chronic hydrocephalus (Skjolding et al. 2013; Trillo‐Contreras et al. 2021) likely represents a compensatory response to persistent CSF imbalance rather than a primary pathogenic mechanism. This compensatory upregulation may indicate that the remaining functional AQP4 channels are working at maximum capacity to maintain whatever CSF clearance is still possible. While chronic hydrocephalus shows increased AQP4 expression, this may not translate to proportionally increase functional water transport due to (1) altered subcellular localization and (2) changes in membrane organization. Supporting this complexity, Ding et al. (2019) demonstrated that preventing AQP4 redistribution (rather than simply increasing expression) in mice attenuated hydrocephalus 28 days after germinal matrix hemorrhage. This emphasizes the pivotal role of AQP4 in hydrocephalus and suggests that effective therapeutic interventions must target both AQP4 expression and its functional organization. Our MR approach captures long‐term genetic effects on AQP4 function but cannot distinguish between acute pathogenic effects and chronic compensatory responses. Further research incorporating temporal analysis of AQP4 expression, localization, and functional activity across different stages of hydrocephalus development is essential for developing stage‐specific therapeutic strategies.

Recently, two studies have underscored the crucial role of choroid plexus immunity in the pathologies of hydrocephalus (Li et al. 2023; Robert et al. 2023). Besides, choroid plexus hyperplasia is also closely associated with hydrocephalus (Hashimoto et al. 2023; Cox et al. 2016). Interestingly, Kim et al. (2020) demonstrated an increase in choroid plexus volume in NMOSD. However, a larger‐scale study did not show an enlargement of choroid plexus volume in NMOSD compared to the normal population (Müller et al. 2022). The contradictory findings regarding choroid plexus volume in NMOSD (Kim et al. 2020, Cox et al. 2016) may reflect temporal differences in disease assessment. Kim et al. (Hashimoto et al. 2023) examined patients close to acute attacks (median = 11 days), while the larger study (Cox et al. 2016) assessed patients in chronic phases (median = 3.3 years). This suggests choroid plexus changes may be dynamic—initially enlarging during acute inflammation, then normalizing or atrophying in chronic phases due to tissue damage and treatment effects. This mechanistic complexity highlights limitations of our MR approach, which captures average genetic effects rather than dynamic, phase‐specific relationships. Future longitudinal studies across different disease phases will be essential for understanding these temporal dynamics.

Importantly, our bidirectional MR analysis revealed no evidence of a causal effect from hydrocephalus to AQP4‐ab‐positive NMOSD, supporting a unidirectional relationship. This null finding suggests that the presence of hydrocephalus does not increase the risk of initiating or exacerbating AQP4 antibody production. Biologically, this is consistent with current understanding of disease mechanisms: AQP4‐ab‐positive NMOSD is an autoimmune astrocytopathy driven by humoral immune responses targeting astrocytic water channels, whereas hydrocephalus is primarily a mechanical or inflammatory disturbance of CSF circulation. While AQP4 dysfunction may plausibly contribute to CSF dysregulation and hydrocephalus, the reverse pathway—where increased intracranial pressure or ventricular enlargement triggers autoimmune sensitization to AQP4—lacks mechanistic support. Therefore, the absence of reverse causality strengthens the interpretation that immune‐mediated injury in NMOSD may predispose to hydrocephalus, rather than hydrocephalus inducing NMOSD.

Our research has several limitations. First, because the GWASs of exposure and outcome used in our study were all based on the patients with European ancestry, the generalizability of our findings to other ethnicities might be limited. Further research to broaden our findings by incorporating diverse ethnic populations may be crucial. Second, the sample sizes of GWAS data are small. Because the GWAS of both the exposure and outcome traits were based on modest‐sized populations, only a few genome‐wide significant SNPs (3–5 instruments) could be identified. This constraint reduces the ability to detect small‐to‐moderate causal effects and increases the likelihood of false negative results, particularly in the reverse MR analysis. The small GWAS sample sizes warrant cautious interpretation and highlight the need for future replication in larger, multiethnic cohorts. Besides, the small number of instrumental SNPs may introduce weak instrument bias and reduce power to detect modest causal effects. Weak instruments can lead to imprecise causal estimates and an increased risk of false negatives or biased results. To address this, we calculated the *F*‐statistics for all included SNPs, all of which exceeded the conventional threshold of 10, suggesting sufficient instrument strength. Finally, despite conducting numerous sensitivity analyses, we cannot rule out the presence of uncontrolled pleiotropies or heterogeneities.

## 5 Conclusion

Our results indicate that genetically predicted AQP4‐ab‐positive NMOSD significantly increase the risk of hydrocephalus. The reduced immune activity of AQP4 may play an important role in the pathogenies of hydrocephalus, and activating AQP4 may be a promising therapeutic approach for hydrocephalus.

## Author Contributions

**Weitao Zhong**: Conceptualization; Writing — review and editing; Methodology; Data curation; Supervision; Writing — original draft; Investigation; Validation; Formal analysis. **Weisong Li**: Data curation; Investigation; Formal analysis; Visualization; Validation. **Qiwei Huang**: Data curation; Formal analysis; Validation; Investigation. **Zelin Li**: Data curation; Formal analysis; Validation; Investigation. **Qiang Wang**: Writing — review and editing; Formal analysis. **Wangming Zhang**: Methodology; Formal analysis; Writing — review and editing.

## Ethics Statement

Our study only relied on publicly accessible data, with no direct involvement of patients in the overall process. The data sources were approved by the appropriate institutional review boards of the original studies.

## Conflicts of Interest

The authors declare no conflicts of interest.

## Peer Review

The peer review history for this article is available at https://publons.com/publon/10.1002/brb3.70804.

## Data Availability

The exposure GWAS data used for our MR analyses are publicly available from the GWAS catalog (https://www.ebi.ac.uk/). The outcome GWAS data used for our MR analyses are publicly available from the FinnGen R10 (https://r10.finngen.fi/).
